# A Visual Analytic Tool (VIADS) to Assist the Hypothesis Generation Process in Clinical Research: Mixed Methods Usability Study

**DOI:** 10.2196/44644

**Published:** 2023-04-27

**Authors:** Xia Jing, Vimla L Patel, James J Cimino, Jay H Shubrook, Yuchun Zhou, Brooke N Draghi, Mytchell A Ernst, Chang Liu, Sonsoles De Lacalle

**Affiliations:** 1 Department of Public Health Sciences College of Behavioral, Social and Health Sciences Clemson University Clemson, SC United States; 2 Cognitive Studies in Medicine and Public Health The New York Academy of Medicine New York, NY United States; 3 Informatics Institute School of Medicine University of Alabama at Birmingham Birmingham, AL United States; 4 Primary Care Department College of Osteopathic Medicine Touro University Vallejo, CA United States; 5 Department of Educational Studies The Patton College of Education Ohio University Athens, OH United States; 6 Electrical Engineering and Computer Science Russ College of Engineering and Technology Ohio University Athens, OH United States; 7 Health Science Program California State University Channel Islands Camarillo, CA United States

**Keywords:** usability, VIADS, data-driven hypothesis generation, visualization, clinical research, SUS, mixed methods study

## Abstract

**Background:**

Visualization can be a powerful tool to comprehend data sets, especially when they can be represented via hierarchical structures. Enhanced comprehension can facilitate the development of scientific hypotheses. However, the inclusion of excessive data can make visualizations overwhelming.

**Objective:**

We developed a visual interactive analytic tool for filtering and summarizing large health data sets coded with hierarchical terminologies (VIADS). In this study, we evaluated the usability of VIADS for visualizing data sets of patient diagnoses and procedures coded in the International Classification of Diseases, Ninth Revision, Clinical Modification (ICD-9-CM).

**Methods:**

We used mixed methods in the study. A group of 12 clinical researchers participated in the generation of data-driven hypotheses using the same data sets and time frame (a 1-hour training session and a 2-hour study session) utilizing VIADS via the think-aloud protocol. The audio and screen activities were recorded remotely. A modified version of the System Usability Scale (SUS) survey and a brief survey with open-ended questions were administered after the study to assess the usability of VIADS and verify their intense usage experience with VIADS.

**Results:**

The range of SUS scores was 37.5 to 87.5. The mean SUS score for VIADS was 71.88 (out of a possible 100, SD 14.62), and the median SUS was 75. The participants unanimously agreed that VIADS offers new perspectives on data sets (12/12, 100%), while 75% (8/12) agreed that VIADS facilitates understanding, presentation, and interpretation of underlying data sets. The comments on the utility of VIADS were positive and aligned well with the design objectives of VIADS. The answers to the open-ended questions in the modified SUS provided specific suggestions regarding potential improvements for VIADS, and the identified problems with usability were used to update the tool.

**Conclusions:**

This usability study demonstrates that VIADS is a usable tool for analyzing secondary data sets with good average usability, good SUS score, and favorable utility. Currently, VIADS accepts data sets with hierarchical codes and their corresponding frequencies. Consequently, only specific types of use cases are supported by the analytical results. Participants agreed, however, that VIADS provides new perspectives on data sets and is relatively easy to use. The VIADS functionalities most appreciated by participants were the ability to filter, summarize, compare, and visualize data.

**International Registered Report Identifier (IRRID):**

RR2-10.2196/39414

## Introduction

Data visualization, especially when data sets can be represented via hierarchical structures of biomedical terminology, has unique and superior advantages for human comprehension over other data presentation formats, such as tables and text [1]. However, the size of a visualization matters, as too much information can still be overwhelming even in this format [2-4]. Therefore, visualization alone may not be adequate to facilitate human comprehension. Instead, visualizing optimal sizes and complexity provides the desired enhancement to human comprehension of the underlying data sets.

Our visual interactive analytic tool for filtering and summarizing large health data sets coded with hierarchical terminologies (VIADS) is a secondary data analysis tool capable of providing visualization, filtering, analysis, summation, and comparison of data sets derived from the International Classification of Diseases, Ninth Revision, Clinical Modification (ICD-9-CM) [5]; the International Classification of Diseases, Tenth Revision, Clinical Modification (ICD-10-CM) [6]; or the National Library of Medicine’s list of Medical Subject Headings (MeSH) [7] and their usage frequencies [8,9]. With existing ICD-9-CM codes, including diagnosis and procedure codes, and the steadily accumulating ICD-10-CM codes, numerous institutions and practices have data sets that VIADS can utilize. Meanwhile, PubMed continues to accumulate MeSH usage data, which VIADS can also use. By exploring summary views of underlying data sets or comparisons of similar data sets via VIADS, users can obtain overviews of data sets and highlights of the differences between the underlying data sets, which may aid in resource allocation decisions or comparisons of different but similar procedures or medications and their associated effects. In clinical research, the latter can facilitate hypothesis generation and validation. These are 2 typical VIADS use cases, one for health care administrators and the other for clinical researchers.

Our team developed the underlying algorithms and threshold settings for filtering and displaying such data sets using example applications. Additionally, we developed a free, publicly accessible web-based version of the tool for educational and research purposes [4,8-11]. Furthermore, VIADS can filter data sets by tuning thresholds to keep and present the most crucial data based on frequencies; visualizing results; comparing similar data sets (eg, data from 2005 versus 2015 or data between 2 hospitals); highlighting differences between data sets (ie, the most statistically significantly different ICD-9 codes between the 2 data sets); and summarizing results (ie, the aggregated results and displayed in the more generic and upper-level categories of the ICD-9 code system) using hierarchical terminologies, codes, and usage frequencies. VIADS could provide visualization (eg, the ICD-9 hierarchical structure, bar charts, and 3D plots) and interactive features (eg, when a user hovers the mouse on a node, more detailed information about that node in the data set will be provided; zoom in; various horizontal spacing layout options; select an algorithm and set thresholds accordingly) to assist users in determining thresholds when using VIADS to generate graphs. The comparative summary provided by VIADS compares 2 data sets. It displays the results in a single visualization, highlighting statistically significant differences (ie, ICD-9 codes) between the 2 data sets. Other research groups have recognized the unique value of visualizing hierarchical structures and have explored such relationships in medicine, social media, and information security [12-18].

In order to evaluate the usability and utility of VIADS, we designed and conducted a study to examine the process of generating clinical research hypotheses by clinical researchers with varying levels of experience (ie, the use case of VIADS by clinical researchers). This consisted of 2 groups of participants who used VIADS and 2 groups who did not. In each study session, all study groups used the same data sets (ie, ICD-9-CM diagnostic and procedural codes with frequencies) and the same time frame to generate data-driven hypotheses in the clinical research context [19]. The hypothesis generation process refers to the process researchers use to generate hypotheses. Some are data-driven, such as the process we used in the study session to generate hypotheses based on the data analysis results and visualization; others are observational-based, such as the unusual phenomena observed during wet lab experiments and the process between observing the phenomena and forming a hypothesis based on the phenomena.

The primary purposes of the study included the identification of (1) the potential role of VIADS in the generation of clinical research hypotheses, (2) the process of hypothesis generation in the context of clinical research, and (3) the role of experience level and its impact on the process of hypothesis generation. In this manuscript, we examine the usability of VIADS. We aimed to disseminate this VIADS usability study’s methods and findings to provide insight into the user interface design of secondary data analysis tools such as VIADS. We hope our experience will aid in the design and development of future data analysis software.

## Methods

### Methods for the Usability Study of VIADS

In this study, we used mixed methods. Participants in this study used VIADS for the hypothesis generation process. For this study, we modified the System Usability Scale (SUS; Multimedia Appendix 1) survey to assess the usability of VIADS. Brooke first proposed the SUS [20,21] in 1996, and it has been widely used to assess the usability of information systems for decades [22-25]. We modified the SUS by including open-ended questions that elaborate and clarify the Likert scale options. For example, if a user selected “disagree” or “strongly disagree” in response to the statement “I think VIADS is easy to use,” a follow-up question asked, “Can you please give an example of how VIADS is not easy to use?” This provided more specific feedback and determined why responses to specific items were unfavorable. The primary objective of this evaluation was to identify improvement opportunities for VIADS. Without explaining the respondent’s score selection, the SUS scores, in our opinion, lacked significant meaning. After the SUS evaluation, VIADS could be enhanced if some negative feedback could be addressed. As a result, we modified the standard SUS (ie, the follow-up questions can assist us in identifying areas that require improvement). Only negative responses were accompanied by a request for clarification.

### Utility Component of VIADS

We administered a 6-question follow-up survey at the end of the study to verify the VIADS usage experience with possible responses of “yes,” “maybe,” “no,” and ”Please elaborate on your answers” (open-ended, optional). Of the 6 questions, 1 question pertained to the overall usefulness of VIADS in clinical research, while the remaining 5 pertained to the specific ways in which VIADS could contribute to the research process. These questions focused on their perception of capacity to (1) provide novel perspectives, (2) facilitate data presentation, (3) facilitate results interpretation, (4) facilitate decision-making, and (5) facilitate other aspects of research. These questions are primarily aligned with the VIADS functionality, with the VIADS design objectives. These are subjective VIADS utility measurements; however, the answers are based on their 1-hour training and 2-hour intense use of VIADS. The objective measures of the utility of VIADS, such as a comparison of the quality of hypotheses generated via VIADS and without VIADS, are currently ongoing and will be shared with readers in separate manuscripts. The cognitive process analysis of the recorded think-aloud sessions is ongoing and will be published separately.

This usability evaluation study was conducted while the participants implemented the think-aloud technique with identical data sets to generate data-driven hypotheses using VIADS. All participants in the study adhered to the same protocol (Multimedia Appendix 2). Multimedia Appendix 3 contains the data extracted from the National Ambulatory Medical Care Survey (NAMCS) conducted by the Centers for Disease Control and Prevention [26,27]. We used data collected in 2005 and 2015 and preprocessed the NAMCS data sets by calculating and aggregating the ICD-9-CM diagnostic and procedural codes and their frequencies. VIADS accepts files in CSV format with 2 columns, one containing ICD-9 codes and the other containing the aggregated ICD-9 code frequencies. The same researcher conducted each study session remotely (via WebEx video conference).

Each participant had a 1-hour training session (Multimedia Appendix 4 contains the training slides that outline the primary functionalities and algorithms of VIADS) followed by a 2-hour study session. In each study session, a participant used the same data sets to perform the analysis; based on his or her experience and knowledge as well as the analysis results, hypotheses were generated, were recorded, and are currently being evaluated by an expert panel. An example of data analysis would be to examine the most frequently used ICD-9 codes in 1 year (2005 or 2015) or to compare the change in ICD-9 code frequencies between 2005 and 2015. During the study session, however, no particular algorithms were requested; each participant was free to explore any algorithms they desired. During the training sessions, the most commonly used scenarios of VIADS were demonstrated to each participant by the researcher. The results reported in this manuscript are based on the participants’ evaluations after the study sessions, which were recorded using BB FlashBack [28] to capture screen activities and conversations between each participant and the researcher. A professional transcription service subsequently transcribed the audio recordings. The modified SUS and an additional follow-up survey containing the 6 questions were administered after each study session. Participants were compensated based on their time spent on the study. Multimedia Appendix 5 is a VIADS user manual with additional information on how to use VIADS specifically.

The data-driven hypothesis generation process results are currently being encoded and analyzed. Once this step is complete, the results will be made public. Therefore, the quality of the hypotheses and the actual cognitive processes involved in hypothesis generation during each study session will be published separately.

### Ethics Approval

The institutional review boards of Clemson University (IRB2020-056) and Ohio University (18-X-192) approved the study. All consent forms and study scripts were shared with all participants prior to the study sessions. The study data sets were shared with each participant on the day of the study session. Verbal permissions were obtained before the study sessions were recorded with each participant.

## Results

### Overview of Results

VIADS was tested by 12 participants, all clinical researchers. They were recruited through multiple national platforms, such as the American Medical Informatics Association discussion forums. Therefore, they were from geographically diverse institutions. Table 1 shows the demographic characteristics of the study participants.

**Table 1:** Demographic characteristics of participants in the usability evaluation of the visual interactive analytic tool for filtering and summarizing large health data sets coded with hierarchical terminologies (VIADS; n=12).

Characteristics			Results, n
**Gender**			
	Female	5	
	Male	7	
**Age group (years)**			
	<35	6	
	35-45	2	
	46-55	4	
**Experience in clinical research (years)**			
	<2	6	
	2-5	3	
	5-10	3	
**Specialties**			
	Health science	3	
	Internal medicine	3	
	Neurology	1	
	Pharmacy	2	
	Primary care	1	
	Other	2	

### SUS Results for VIADS

Table 2 shows the SUS scores for each participant. Among the 12 participants, 2 had SUS scores <60, and 5 had SUS scores ≥80. The scores ranged from 37.5 to 87.5. The overall mean SUS score for VIADS was 71.88 (SD 14.62), and the overall median SUS score was 75.

Table 3 presents the detailed raw SUS evaluation results for VIADS without SUS calculations. It summarizes the raw evaluation scores for each SUS evaluation item, with the following range of scores: strongly disagree=1 to strongly agree=5. For one-half of the questions in SUS, higher scores denoted more positive responses (direct questions); for the other one-half, lower scores indicated more positive responses (reverse questions).

The mean results for the direct questions ranged from 3.75 to 4.25 out of 5. The median score for all direct questions was 4. The scores for the reverse questions ranged from 1.92 to 2.83. For the reverse questions, 4 median scores were 2, and 1 median score was 3.

**Table 2:** System Usability Scale (SUS) scores for the visual interactive analytic tool for filtering and summarizing large health data sets coded with hierarchical terminologies (VIADS) from the individual participants (n=12).

Participant number	SUS score
P1	82.5
P2	85
P3	67.5
P4	72.5
P5	55
P6	65
P7	80
P8	85
P9	77.5
P10	37.5
P11	87.5
P12	67.5

**Table 3:** Detailed System Usability Scale (SUS) evaluation items and raw scores (n=12).

SUS item^a^	Maximum score	Minimum score	Mean score	Median score
Would use frequently^b^	5	3	3.75	4
Unnecessarily complex^c^	4	1	2.33	2
Easy to use^b^	5	1	4.17	4
Need tech support to use^c^	4	1	2.50	2
Integrated well^b^	5	2	3.83	4
Inconsistencies^c^	3	1	1.92	2
Learned to use VIADS^d^ quickly^b^	5	1	4.00	4
Cumbersome to use^c^	3	1	1.75	2
Can use confidently^b^	5	2	4.25	4
Need to learn more^c^	4	2	2.83	3

^a^Strongly disagree=1; strongly agree=5.

^b^Higher scores are favorable.

^c^Lower scores are favorable.

^d^VIADS: visual interactive analytic tool for filtering and summarizing large health data sets coded with hierarchical terminologies.

### Utility Survey Results for VIADS

The modified SUS questionnaire and utility questions were asked and answered after a 1-hour training session and a 2-hour study session; when matched to the SUS scores, their answers corroborated their positive usage experience of VIADS. Table 4 presents the results of our VIADS utility questions. As indicated in Table 4, all results were separated into 3 categories: “Yes,” “Maybe,” or “No.” Among the respondents, 100% (12/12) agreed (ie, they all selected Yes) that VIADS provides new perspectives on the underlying data sets, 92% (11/12) felt that it could facilitate the presentation of data sets, and 75% (8/12) agreed that VIADS is a valuable tool for clinical research. Additionally, 75% (8/12) agreed that VIADS could facilitate the interpretation of results and decision-making in hypothesis generation. More than one-half (7/12, 58%) of the participants expressed conservative attitudes when asked if VIADS could assist with other aspects of research (ie, 58% selected either “maybe” or “no” as answers). In addition to subjective measures of the utility of VIADS, we published some objective measures at a conference [29]. For example, participants could generate 5 to 21 hypotheses within 2 hours, and the VIADS group took a shorter time, on average, to generate each hypothesis when we did not consider the quality of the hypotheses. More objective measures (such as the quality of the hypotheses) are still under analysis.

**Table 4:** The visual interactive analytic tool for filtering and summarizing large health data sets coded with hierarchical terminologies (VIADS) utility questions and results (n=12).

VIADS utility survey item	Yes, n (%)	Maybe, n (%)	No, n (%)
Provides new perspectives or measurements for data sets	12 (100)	0 (0)	0 (0)
Facilitates the interpretation of data sets	9 (75)	2 (17)	1 (8)
Facilitates decision-making in hypothesis generation	9 (75)	3 (25)	0 (0)
Facilitates the presentation of data sets	11 (92)	1 (8)	0 (0)
Useful in additional aspects of research	5 (42)	6 (50)	1 (8)
A useful tool for research overall	9 (75)	3 (25)	0 (0)

### Qualitative Results From Open-ended Questions

Specific comments on answers to open-ended questions were organized as positive comments and suggestions, some of which were not positive. All positive comments were categorized under thematic headings, and only up to 3 items were presented in Table 5. The themes emerged after we aggregated and synthesized all comments from participants.

The following insights for the improvement of VIADS were answers to the open-ended questions included in the modified SUS: (1) label data sets during comparison and carry the data set labels across pages, (2) more tips to explain the settings while uploading the data sets, (3) include the definitions of the terms and parameters used in VIADS, (4) the data sets accepted by VIADS are very specific, (5) provide further elaboration on the error messages, (6) provide a more detailed description of the functions.

**Table 5:** Thematic headings for the open-ended questions and examples for each theme.

Thematic heading	Example statements
VIADS^a^ facilitates the visualization of data sets to enhance understanding.	“Pictorial and easy to read and understand huge data sets.” “VIADS presents a large data set containing diagnoses codes in an organized, intuitive graphical output with simple summary statistics that can be interpreted quickly and at a summary level, allowing for better understanding of the data set and how it can be analyzed.” “I think that VIADS would help with methodology, analysis, descriptive statistics, and presentation of results as well.”
VIADS provides a comparison function that compares similar data sets and highlights the results.	“Comparison of complex data sets would be easy with this type of visualization.” “It is nice to have comparison of data sets, but that also goes back to understanding what the data set consists of.” “By comparing different sets of data, it would help clarify if it is an important/relevant area to study.”
The filtering function is a helpful means of reducing the size of data sets easily and effectively.	“By being able to utilize large sets of data and recognize top percentages or number of certain topics, it helps you focus on an area to potentially study.“ “I think the VIADS system is really cool, and it’s fascinating how it operates. I would say that it’s a great tool with algorithms in place but can also be overwhelming sometimes if there is overabundance of data. What’s great is that we can reduce the amount of data displayed. I think the fine tuning of threshold can be tricky though and difficult if we don’t understand what CC^b^ or NC^c^ or CC + ratio means.”
VIADS facilitates thought processes and hypothesis generation.	“The many results and branches definitely help generate hypotheses. In the beginning, it is a little difficult since I would be focused on how to sort the data or minimize how many nodes/results are displayed, but after a while, with the key terms and diagnoses, it triggers my thought process so I think it could help with generating new ideas.” “VIADS answered many of the questions I had about the data set before using the tool. After using VIADS, I felt that some of my hypotheses would be valuable to pursue and some would not be as much. It also helped me build on some initial hypotheses to generate more specific and advanced questions.” “At least this session, the amount of diagnoses present and how it branches from one another helps not only stir up thoughts of known studies or information but helped me think of new ones or new questions that may not be answered yet.”
Other useful features of VIADS	“I think that VIADS is very useful because it is simple and has a specific purpose that it serves well. I have used ICD codes frequently in my work so far, and I think this type of specific tool would be helpful for many applications.” “I think the training session helps a lot, because if I were to navigate it on my own, I probably wouldn’t know what the branching from each category means and the nodes when I did a preview, but it helps to click around.” “The graphical output and ability to export the diagram would be very useful in presenting diagnosis data.”
Suggestions	“Not confident enough to base my hypothesis on numbers only.” “I think it could but then I would need training on how to create a data set to meet the VIADS system or how to set up complex data on a spreadsheet so when it’s uploaded to VIADS, it can help sort.” “Just that the buttons need to be adjusted...they don’t always ‘click’ unless you put your mouse on a very specific spot.”

^a^VIADS: visual interactive analytic tool for filtering and summarizing large health data sets coded with hierarchical terminologies.

^b^CC: class count.

^c^NC: node count.

## Discussion

### Interpretation of the Results

Previous research indicates that the mean SUS usability score is 68, on average, regardless of specific applications (eg, information systems or apps) [22]. The mean SUS score for VIADS in this study was 71.88, and the median score was 75. The literature shows that these are good usability scores [23,30]. Although the average score for VIADS can be improved further, it should be noted that VIADS is a complex analytic tool with many functionalities. The SUS score was encouraging, given the complexity of VIADS and participants' heterogeneous backgrounds. Only 2 of the 12 participants had SUS scores <60. The rest had scores ≥65, and 5 had SUS scores ≥80. Table 2 includes the SUS score for each participant in the VIADS group. Furthermore, the additional questions and constructive insights to improve the VIADS interface and instructions will help us to address these concerns more explicitly.

The average SUS score was 71.88, with an SD of 14.62, which is approximately 20% of the mean SUS. This large SD indicates heterogeneous opinions among participants about the usability of VIADS, allowing us to make more prudent and selective decisions about revisions to VIADS rather than implementing all suggestions. It is possible to investigate the variables contributing to such heterogeneity in a larger sample.

The feedback on the utility of VIADS was predominantly and consistently positive. The follow-up survey results provided some degree of the utility of VIADS, especially after 1 hour of training and 2 hours of using VIADS to analyze the data and generate hypotheses. As a secondary data analytic tool, VIADS fulfills its design purposes. All participants agreed that VIADS offers new perspectives and measures of data sets. The usefulness of VIADS in facilitating data presentation (11/12, 92%), results interpretation, and decision-making in hypothesis generation was agreed upon by at least 75% of the participants. There appeared to be some reservations among the participants about making positive statements on additional aspects of clinical research beyond the dimensions about which they were explicitly asked. However, this could suggest that participants were only prepared to respond to items about which they felt sure. Therefore, we could take these results as additional validation of the positive nature of the overall results, acknowledging that there is always room for improvement.

Among all suggestions to improve VIADS among participants, suggestions 1 (ie, label data sets during comparison and carry the data set labels across pages), 2 (ie, more tips to explain the settings while uploading the data sets), and 5 (ie, provide further elaboration on the error messages) can be added to the VIADS interface. Suggestions 3 (ie, include the definitions of the terms and parameters used in VIADS) and 6 (ie, provide a more detailed description of the functions) are provided in the VIADS user manual and may be highlighted. There is also a legend key in the main interface. Furthermore, point 4 (ie, the data sets accepted by VIADS are very specific) is a limitation of VIADS; although the revisions are ongoing for all other points, point 4 has been excluded. To address point 4, a new tool is needed, which is under development.

Most of the participants positively commented on specific aspects of VIADS. However, it is possible that participants who provided lower SUS ratings were less inclined to leave comments on specific features.

**Figure 1:**
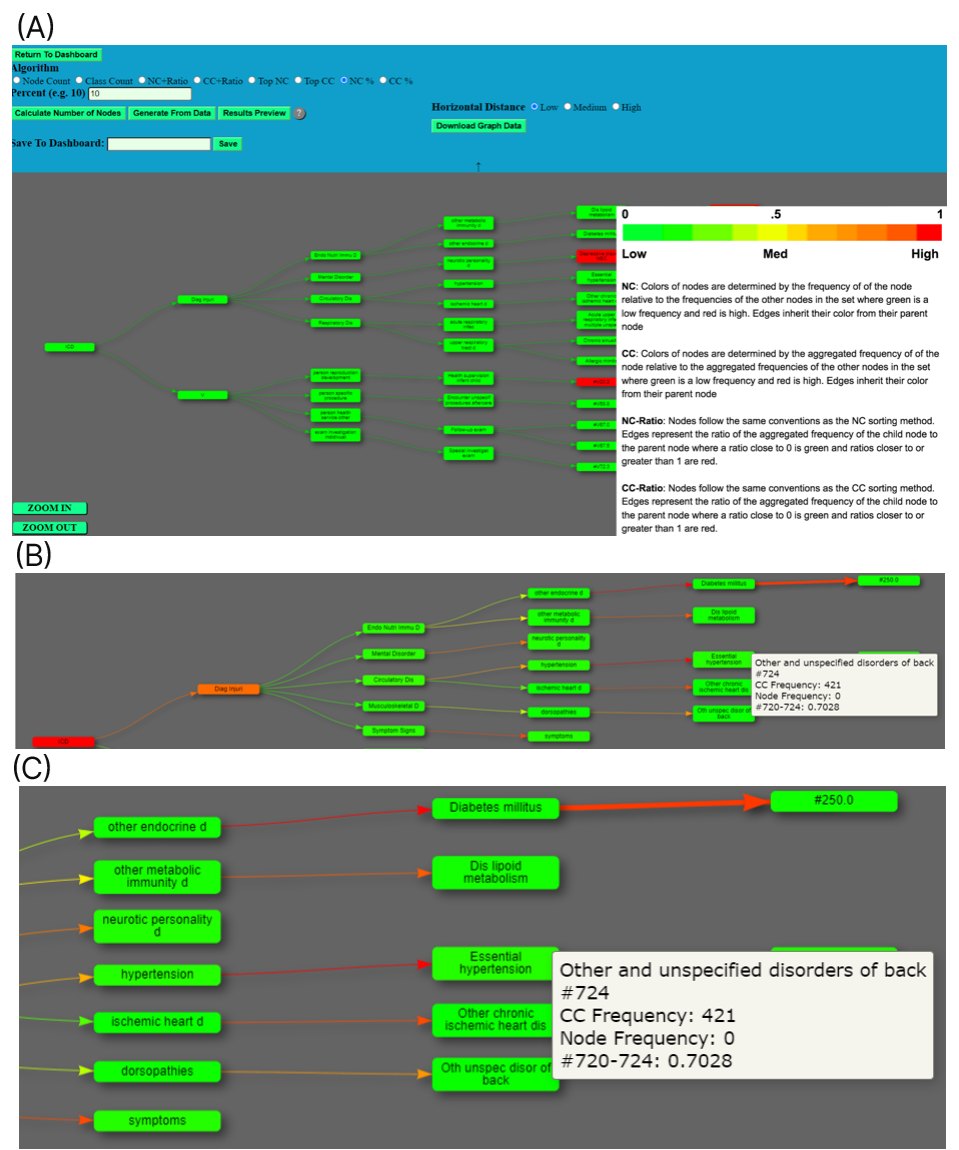
(A) Screenshot of the visual interactive analytic tool for filtering and summarizing large health data sets coded with hierarchical terminologies (VIADS) showing the algorithms, generated graph, terms used in the interface, and definitions provided by VIADS; (B, C) enlarged portions of a graph generated by VIADS.

In a system such as VIADS, it can be challenging to balance usability and utility. The functionality of the tool is not simple, and users must understand the underlying algorithms and how to use the tool’s various features and interpret the results it generates. The terms used in the interface alone (eg, NC is for node count, and CC is for the class count) represent a long list of definitions for users to grasp (Figure 1). The comparison summary of VIADS is presented using a single visualization (ie, the ICD-9 hierarchical structure), with highlighted ICD-9 codes if they are statistically different between the 2 data sets. During the development of VIADS, we devoted considerably more time to the utility of the tool, in terms of implementing the desired functionalities, than to the interface’s usability. Although we are encouraged by the SUS scores and the participants’ acclaim for VIADS’s primary features, which is their perception after their intense use of VIADS (ie, 1-hour training and 2 hours of use), actual performance measures are needed and ongoing.

Think-aloud protocols have been used as a method in the evaluation of information systems for decades. Some studies have focused on the investigations of the medical reasoning process [31-35], evaluation of clinical decision support systems [30,36,37], and additional purposes [25,38,39]. Our study used a think-aloud protocol to access the researchers’ thoughts, while participants used VIADS to assess its usability and utility.

### Significance of the Work

We asked the general research question: “Can secondary data analytic tools, such as VIADS, facilitate the hypothesis generation process?” One aspect of the tool related to this question is its usability. Thus, our objective was to investigate the tool’s usability and utility using mixed methods. The process of generating hypotheses using the same data sets via VIADS for clinical research projects was used as a task by participants, which provided real-use experience before participants answered the SUS and utility surveys. The results show the tool’s usability and some degree of utility. VIADS can be constantly updated with users’ feedback. This is an important first step to exploring the role of VIADS in facilitating clinical researchers to generate research and scientific hypotheses and support them at various levels of research.

Furthermore, this useful and accessible tool is freely available online as a user-friendly version, allowing users to leverage the tool without investing unnecessary time in technical details. Our research established a link between using a secondary data analysis tool and facilitating scientific hypothesis generation. This can be a starting point for utilizing secondary data analysis tools to understand the cognitive process of scientific hypothesis generation better.

### Strengths and Limitations of This Study

The study included 12 participants, above the average range for a usability study. Past studies showed that 5 [38], 7 [30], 8 [36], and 12 [37] participants participated in comparable usability studies. The literature indicates that 5 participants can identify approximately 55% of usability issues, while 10 can identify approximately 80% [40]. With 12 participants, we are relatively confident that our usability study has a sufficient number of participants. In addition, our participants were selected from different regions of the country, with varying backgrounds within the clinical research context, providing a more comprehensive perspective of the tool.

Our SUS modification allowed participants to elaborate on the scores assigned to each SUS item. This allowed for targeted VIADS revisions. We believe that our modifications to the SUS were valuable and beneficial additions to the original SUS survey. Despite being grounded in the actual functionality of VIADS, the 6 utility questions and the SUS questions aligned well with the Health Information Technology Usability Evaluation Scale (Health-ITUES) [41]. In terms of health technology assessment frameworks [42,43], VIADS more closely resembles a data analysis tool than a mobile health application. Therefore, the economic evaluation of the tool’s impact deviates slightly from the tool’s primary purpose.

However, we know VIADS accepts only very specific types of data sets, not all. Consequently, the conclusions drawn from the data sets are specific rather than general. Now, we are developing a more generic supporting tool with a broader range of support for researchers.

Question 5 in Table 4 has the lowest agreeable rate; only 42% (5/12) of participants selected “Yes,” and 50% (6/12) selected “Maybe.” This question was supposed to capture any unintended impact of VIADS in addition to the 4 intended functionalities (ie, questions 1 to 4 in Table 4). However, the current presentation of the question can be confusing, which may lead to a low agreeable rate.

We recognize that our usability testing tool (SUS) captures the users’ perceptions, not how VIADS was used. Even though each participant had an intense VIADS use session before they completed the SUS survey, this still is a limitation of this study.

Due to lack of expertise, the graphs generated by VIADS consider more of the meanings and align with the underlying algorithms of VIADS, without much consideration of artistical aspects or color-blind users. Therefore, this is another limitation of this study. Even though there is no specific feedback on the artistic aspects of VIADS, this can be an area for improvement with appropriate additional expertise in the future.

### Future Directions

We aim to increase the impact of VIADS through the (1) promotion of VIADS to increase its visibility among potential users and (2) development of new applications that facilitate the integration of VIADS with electronic health record systems or data repositories. This will enable VIADS to function as an add-on to existing systems that host large amounts of patient data. Through its analytical and visualization capabilities, the integrated version will streamline data sources, thereby promoting the adoption and use of the tool. Increasing the number of terminologies supported by VIADS is another possible area for further investigation. Finally, we could evaluate the tool at various stages and continuously use an iterative design process to improve VIADS.

### Conclusion

VIADS, a tool that facilitates the generation of hypotheses in clinical research contexts, is a valuable addition to existing secondary data analysis tools. After intense use sessions, a diverse sample of clinical researchers perceived it to be useful and relatively usable. The new perspectives on hierarchical data sets and an easy-to-use interface provided by VIADS were recognized by users. The availability and use of ICD-9-CM, ICD-10-CM, and MeSH-coded data sets enable practical and convenient comparison of data sets and have many potential health care applications.

## Appendix

Multimedia Appendix 1 Modified SUS survey (with utility questions) for evaluation of VIADS.

Multimedia Appendix 2 Study script used for VIADS usability and utility study.

Multimedia Appendix 3 Data sets used to conduct usability and utility study of VIADS.

Multimedia Appendix 4 Training materials used during the training session for VIADS.

Multimedia Appendix 5 VIADS user manual.

## Abbreviations

Health-ITUES: Health Information Technology Usability Evaluation Scale
ICD-9-CM: International Classification of Diseases, Ninth Revision, Clinical Modification
ICD-10-CM: International Classification of Diseases, Tenth Revision, Clinical Modification
MeSH: Medical Subject Headings
NAMCS: National Ambulatory Medical Care Survey
SUS: System Usability Scale
VIADS: visual interactive analytic tool for filtering and summarizing large health data sets coded with hierarchical terminologies
