# Supplementary material for: A Visual Analytic Tool (VIADS) to Assist the Hypothesis Generation Process in Clinical Research: Mixed Methods Usability Study
Source: JMIR Hum Factors. 2023 Apr 27;10:e44644. doi: 10.2196/44644 (PMC10176142; doi:10.2196/44644)
Supplement: Multimedia Appendix 1 [file humanfactors_v10i1e44644_app1.pdf]

VIADS system usability scale (SUS) and utility questionnaire

Start of Block: Default Question Block

Q1 I would like to use VIADS frequently.

- ◯ Strongly disagree (1)
- ◯ Disagree (2)
- ◯ Neutral (3)
- ◯ Agree (4)
- ◯ Strongly agree (5)

Display This Question:

If I would like to use VIADS frequently. = Strongly disagree

Or I would like to use VIADS frequently. = Disagree

Q2 Can you specify the reasons for your answer (i.e., why will not you use VIADS frequently)?

____

____

____

____

____

Q3 I find VIADS unnecessarily complex.

- ◯ Strongly disagree (1)
- ◯ Disagree (2)
- ◯ Neutral (3)
- ◯ Agree (4)
- ◯ Strongly agree (5)

Display This Question:

If I find VIADS unnecessarily complex. = Strongly agree

Or I find VIADS unnecessarily complex. = Agree

Q4 Please specify what you would do to make VIADS simpler. Please list three things you would change in VIADS if possible.

____

____

____

____

____

Q5 I think VIADS is easy to use.

- ◯ Strongly disagree (1)
- ◯ Disagree (2)
- ◯ Neutral (3)
- ◯ Agree (4)
- ◯ Strongly agree (5)

Display This Question:

If I think VIADS is easy to use. = Strongly disagree

Or I think VIADS is easy to use. = Disagree

Q6 Can you please give an example about VIADS is not easy to use?

____

____

____

____

____

Q7
I would need technical support in order to use VIADS.

- ○ Strongly disagree (1)
- ○ Disagree (2)
- ○ Neutral (3)
- ○ Agree (4)
- ○ Strongly agree (5)

Display This Question:

If I would need technical support in order to use VIADS. = Strongly agree

Or I would need technical support in order to use VIADS. = Agree

Q8 Can you please specify the support (such as navigation, definitions of the terms used in VIADS interfaces, mechanism of VIADS behind the scenes) you need?

____

____

____

____

____

Q9 I find the various functions in VIADS are well integrated with each other.

- Strongly disagree (1)
- Disagree (2)
- Neutral (3)
- Agree (4)
- Strongly agree (5)

Display This Question:

If I find the various functions in VIADS are well integrated with each other. = Strongly disagree

Or I find the various functions in VIADS are well integrated with each other. = Disagree

Q10
Can you please give an example to elaborate your answer? So we can try to look for ways to improve. Please feel free to add suggestions to make them better integrated, ideally.

____

____

____

____

____

Q11 I think there are too many inconsistencies in VIADS.

- ◯ Strongly disagree (1)
- ◯ Disagree (2)
- ◯ Neutral (3)
- ◯ Agree (4)
- ◯ Strongly agree (5)

Display This Question:

If I think there are too many inconsistencies in VIADS. = Strongly agree

Or I think there are too many inconsistencies in VIADS. = Agree

Q12 Can you please give examples of inconsistencies in VIADS?

____

____

____

____

____

Q13 I imagine that most people can learn to use VIADS quickly.

- ◯ Strongly disagree (1)
- ◯ Disagree (2)
- ◯ Neutral (3)
- ◯ Agree (4)
- ◯ Strongly agree (5)

Display This Question:

If I imagine that most people can learn to use VIADS quickly. = Strongly disagree

Or I imagine that most people can learn to use VIADS quickly. = Disagree

Q14 Can you please explain why you think most people cannot learn to use VIADS quickly?

____

____

____

____

____

Q15 I find VIADS cumbersome to use.

- Strongly disagree (1)
- Disagree (2)
- Neutral (3)
- Agree (4)
- Strongly agree (5)

Display This Question:

If I find VIADS cumbersome to use. = Strongly agree

Or I find VIADS cumbersome to use. = Agree

Q16 Can you please give an example that VIADS is cumbersome to use?

____

____

____

____

Q17 I feel confident using VIADS.

- Strongly disagree (1)
- Disagree (2)
- Neutral (3)
- Agree (4)
- Strongly agree (5)

Display This Question:

If I feel confident using VIADS. = Strongly disagree

Or I feel confident using VIADS. = Disagree

Q18 Can you please explain why you are not confident using VIADS?

Q19 I need to learn more before I can use VIADS.

- ○ Strongly disagree (1)
- ○ Disagree (2)
- ○ Neutral (3)
- ○ Agree (4)
- ○ Strongly agree (5)

Display This Question:

If I need to learn more before I can use VIADS. = Strongly agree

Or I need to learn more before I can use VIADS. = Agree

Q20 Can you please specify what you need to learn before you can use VIADS? An example will be very helpful.

____

____

____

____

____

Q21 Does VIADS provide new perspectives or measurements for understanding the data set?

- ○ Yes (1)
- ○ Maybe (2)
- ○ No (3)

Display This Question:

If Does VIADS provide new perspectives or measurements for understanding the data set? = Yes

Or Does VIADS provide new perspectives or measurements for understanding the data set? = Maybe

Q22 Can you specify your answer to provide more details please?

____

____

____

____

____

Q23 Do you think that VIADS can facilitate the interpretation of the data set?

- Yes (1)
- Maybe (2)
- No (3)

Display This Question:

If Do you think that VIADS can facilitate the interpretation of the data set? = Yes

Or Do you think that VIADS can facilitate the interpretation of the data set? = Maybe

Q24 Can you specify your answer to provide more details please?

____

____

____

____

____

Q25 Do you think that VIADS can facilitate your decision making in generating hypothesis?

- Yes (1)
- Maybe (2)
- No (3)

Q26 Can you specify your answer to provide more details please?

____

____

____

____

____

Q28 Do you think that VIADS can facilitate your capability to present the data set?

- Yes (1)
- Maybe (2)
- No (3)

Display This Question:

If Do you think that VIADS can facilitate your capability to present the data set? = Yes

Or Do you think that VIADS can facilitate your capability to present the data set? = Maybe

Q29 Can you specify your answer to provide more details please?

____

____

____

Q30 Do you think that VIADS can be used in other aspects of research questions in addition to facilitating understanding of data, interpretation of data, presentation of data, decisions on hypothesis generation?

- ○ Yes (1)
- ○ Maybe (2)
- ○ No (3)

Display This Question:

If Do you think that VIADS can be used in other aspects of research questions in addition to facilit... = Yes

Or Do you think that VIADS can be used in other aspects of research questions in addition to facilit... = Maybe

Q31 Can you specify your answer to provide more details please?

____

____

____

____

____

Q32 Do you think that VIADS is a useful tool to facilitate research activities in general?

- ○ Yes (1)
- ○ Maybe (2)
- ○ No (3)

Q33 Do you have any additional suggestions or comments about VIADS?

____

____

____

____

____

End of Block: Default Question Block
