# Supplementary material for: A Visual Analytic Tool (VIADS) to Assist the Hypothesis Generation Process in Clinical Research: Mixed Methods Usability Study
Source: JMIR Hum Factors. 2023 Apr 27;10:e44644. doi: 10.2196/44644 (PMC10176142; doi:10.2196/44644)
Supplement: Multimedia Appendix 3 [file humanfactors_v10i1e44644_app3.pdf]

NAMCS2005_Top137NC	
ICD9 codes	Frequency
4019	1733
V6759	957
V202	866
25000	688
2724	635
4659	535
V5889	461
V6709	453
4739	412
311	404
4779	397
41400	378
V7231	366
V4589	363
3829	348
3669	331
V221	325
30000	319
53081	312
49390	281
2720	278
V700	277
29620	265
7020	265
462	260
6929	256
2449	232
5990	219
31401	217
7061	212
60000	208
34690	204
7242	203
496	200
27800	199
490	197
V6549	191
7840	190
2169	189
71590	184
185	183
3004	183
42731	166
29680	162
7862	153

3659	151
78039	150
78650	147
V990	147
V508	145
7245	144
3804	142
4720	141
78079	141
V7189	141
1739	138
4280	132
5997	131
71596	129
3671	128
7291	128
7804	127
3540	125
7999	125
2859	123
1749	120
7810	120
470	119
71946	119
70219	116
31400	115
78057	115
7231	114
30001	113
7295	112
78609	111
79093	110
3320	106
5920	106
7062	102
30002	101
4619	101
78900	97
V7647	95
340	94
V431	94
4660	93
V720	93
73300	92
4781	90
38181	88
V1083	88

71941	87
25050	86
3003	84
56400	84
71690	83
7820	83
38910	82
78820	82
36500	79
38010	79
7823	79
7140	78
V997	78
37230	77
6953	77
340	76
30981	76
8470	76
V5883	76
V1249	75
7244	74
7962	74
29630	73
5939	73
4439	72
36250	71
78052	70
3674	68
4011	68
4241	67
61610	67
6272	67
7099	66
4139	65
6961	65
37921	64
43491	64
55090	64
7234	64
7821	64
78830	64
486	63
3559	61
3569	61
V583	61

NAMCS2015_Top125NC	
ICD9 code	Frequency
4019	2070
25000	1099
2724	1097
V202	924
V5889	697
V6759	653
V700	565
53081	545
7020	538
30000	523
311	513
70219	499
V431	499
41400	464
V7231	457
42731	442
2169	420
36616	405
27800	404
7061	401
4779	389
4011	384
2449	382
2720	382
V221	373
6929	357
37515	350
49390	348
3669	341
V6709	340
4659	328
185	325
3804	322
V4589	306
7242	306
31401	297
60001	290
3829	286
7840	285
60784	284
71946	282
4739	278
5990	272
79093	257
2382	251

V6700	245
5920	243
60000	236
38181	231
36570	231
71690	226
70909	213
V990	212
7862	211
7295	210
496	206
36511	206
33829	204
7804	203
V1083	200
78079	200
7810	194
71596	192
3051	191
470	188
71941	187
2689	185
7245	183
25050	182
37921	180
7062	178
3899	176
34690	176
78841	174
71590	172
32723	171
462	167
4720	161
30002	161
7231	157
V508	156
2722	156
3671	155
47819	155
78650	154
3659	152
41401	150
36500	149
2859	146
V679	144
78052	144
78900	143

340	137
V2389	137
27801	136
V5869	136
1749	136
7291	135
78820	134
78605	133
78609	132
3540	132
7244	132
6953	129
17391	129
7823	126
7820	126
40390	125
29620	125
2572	124
70211	123
6961	122
34590	117
59970	116
29680	116
7099	115
7851	115
6259	114
78830	113
38910	113
4254	113
31400	113
4280	111
5859	110
V4561	110

ICD9 codes	Abbreviations	Full names
1739	Skin: site unspecified	Skin: site unspecified
17391	other malignant neoplasm of skin unspecified	other malignant neoplasm of skin: site unspecified
1749	malignant neoplasm of female breast: unspecified	malignant neoplasm of female breast: unspecified
185	malignant neoplasm of prostate	malignant neoplasm of prostate
2169	skin benign neoplasm unspecified	skin benign neoplasm: unspecified site
2382	neoplasms of uncertain behavior of skin	neoplasms of uncertain behavior of skin
2449	unspecified hypothyroidism	unspecified hypothyroidism
2500	diabetes no complication	Diabetes mellitus without mention of complication
25000	diabetes no complication	diabetes mellitus without mention of complication: type 2 or unspecified type-not stated as uncontrolled
2505	Diabetes with ophthalmic manifestations	Diabetes with ophthalmic manifestations
25050	diabetes with ophthalmic manifestations	diabetes with ophthalmic manifestations type 2 or unspecified type not stated as uncontrolled
2572	other testicular hypofunction	other testicular hypofunction
2689	unspecified vitamin D deficiency	unspecified vitamin D deficiency
2720	pure hypercholesterolemia	pure hypercholesterolemia
2722	mixed hyperlipidemia	mixed hyperlipidemia
2724	other hyperlipidemia	other and unspecified hyperlipidemia
2780	Overweight and obesity	Overweight and obesity
27800	unspecified obesity	unspecified obesity
27801	morbid obesity	morbid obesity
2859	unspecified anemia	unspecified anemia
2962		Major depressive disorder-single episode
29620	major depressive disorder: single episode unspecified	major depressive disorder: single episode unspecified
29630		Major depressive disorder: recurrent episode unspecified
2968		Other and unspecified bipolar disorders
29680	bipolar disorder: unspecified	bipolar disorder: unspecified
3000	Anxiety states	Anxiety states
30000	anxiety state unspecified	anxiety state: unspecified
30001		Panic disorder without agoraphobia
30002	generalized anxiety disorder	generalized anxiety disorder

3003		Obsessive-compulsive disorders
3004		Dysthymic disorder
3051	tobacco use disorder	tobacco use disorder
30981		Posttraumatic stress disorder
311	depression NEC	depressive disorder: not elsewhere classified
3140	Attention deficit disorder	Attention deficit disorder
31400	ADD no hyperactivity	attention deficit disorder without mention of hyperactivity
31401	ADD hyperactivity	attention deficit disorder with hyperactivity
3272	Organic sleep apnea	Organic sleep apnea
32723	obstructive sleep apnea (adult) (pediatric)	obstructive sleep apnea (adult) (pediatric)
3320		Paralysis agitans
3382	Chronic pain	Chronic pain
33829	other chronic pain	other chronic pain
340	multiple sclerosis	multiple sclerosis
3459	unspecified epilepsy	unspecified epilepsy
34590	epilepsy: unspecified	epilepsy: unspecified without mention of intractable epilepsy
3469	unspecified migraine	unspecified migraine
34690	unspecified migraine	unspecified migraine without mention of intractable migraine without mention of status migrainosus
3540	carpal tunnel syndrome	carpal tunnel syndrome
3559		Mononeuritis of unspecified site
3569		Hereditary and idiopathic peripheral neuropathy: unspecified
36250		Macular degeneration (senile)-unspecified
3650	Borderline glaucoma [glaucoma suspect]	Borderline glaucoma [glaucoma suspect]
36500	unspecified preglaucoma	unspecified preglaucoma
3651	Open-angle glaucoma	Open-angle glaucoma
36511	primary open angle glaucoma	primary open angle glaucoma
3657		
36570		
3659	unspecified glaucoma	unspecified glaucoma
3661	Senile cataract	Senile cataract
36616	senile cataract: nuclear sclerosis	senile cataract: nuclear sclerosis
3669	unspecified cataract	unspecified cataract
3671	myopia	myopia

3674		Presbyopia
37230		Conjunctivitis unspecified
3751	Other disorders of lacrimal gland	Other disorders of lacrimal gland
37515	unspecified tear film insufficiency	unspecified tear film insufficiency
3792	Disorders of vitreous body	Disorders of vitreous body
37921	vitreous degeneration	vitreous degeneration
38010		Infective otitis externa unspecified
3804	ear and mastoid process disease: impacted cerumen	diseases of the ear and mastoid process: impacted cerumen
3818	Other disorders of Eustachian tube	Other disorders of Eustachian tube
38181	dysfunction of eustachian tube	dysfunction of eustachian tube
3829	unspecified otitis media	unspecified otitis media
3891	Sensorineural hearing loss	Sensorineural hearing loss
38910	sensorineural hearing loss: unspecified	sensorineural hearing loss: unspecified
3899	unspecified hearing loss	unspecified hearing loss
4011	benign essential hypertension	benign essential hypertension
4019	essential hypt unspecify	essential hypertension: unspecified
4039	Hypertensive chronic kidney disease:unspecified	Hypertensive chronic kidney disease:unspecified
40390	unspecified hypertensive CKD	unspecified hypertensive chronic kidney disease with chronic kidney disease stage I through stage IV or unpescified
4139		Other and unspecified angina pectoris
4140	Coronary atherosclerosis	Coronary atherosclerosis
41400	chronic ischemic heart d unspecify	chronic ischemic heart disease: unspecified type of vessel- native or graft
41401	coronary atherosclerosis of native coronary artery	coronary atherosclerosis of native coronary artery
4241		Aortic valve disorders
4254	other primary cardiomyopathies	other primary cardiomyopathies
4273	Atrial fibrillation and flutter	Atrial fibrillation and flutter
42731	atrial fibrillation	atrial fibrillation
4280	congestive heart failure: unspecified	congestive heart failure: unspecified
43491		Cerebral artery occlusion-unspecified with cerebral infarction
4439		Peripheral vascular disease-unspecified

4619		Acute sinusitis unspecified
462	acute pharyngitis	acute pharyngitis
4659	unspecified acute respiratory infections	unspecified site of acute respiratory infections
4660		Acute bronchitis
470	deviated nasal septum	deviated nasal septum
4720	chronic rhinitis	chronic rhinitis
4739	unspecified chronic sinusitis	unspecified chronic sinusitis
4779	unspecified upper respir tract D	unspecified cause of the upper respiratory tract disease
4781	Other diseases of nasal cavity and sinuses	Other diseases of nasal cavity and sinuses
47819	other disease of nasal cavity and sinuses	other disease of nasal cavity and sinuses
486		Pneumonia organism unspecified
490		Bronchitis not specified as acute or chronic
4939	unspecified asthma	unspecified asthma
49390	unspecified asthma	unspecified asthma
496	chronic airway obstruction: NEC	chronic airway obstruction: not elsewhere classified
5308	Other specified disorders of esophagus	Other specified disorders of esophagus
53081	esophageal reflux	esophageal reflux
55090		Inguinal hernia without mention of obstruction or gangrene unilateral or unspecified
56400		Constipation unspecified
5859	chronic kidney disease: unspecified	chronic kidney disease: unspecified
5920	calculus of kidney	calculus of kidney
5939		Unspecified disorder of kidney and ureter
5990	urinary tract infection unspecified	urinary tract infection: stie not specified
5997	Hematuria	Hematuria
59970	hematuria: unspecified	hematuria: unspecified
6000	Hypertrophy (benign) of prostate	Hypertrophy (benign) of prostate
60000	hypertrophy (benign) of prostate without urinary obstruct-LUTS	hypertrophy (benign) of prostate without urinary obstruction and other lower urinary tract sympotoms (LUTS)
60001	hypertrophy (benign) of prostate with urinary obstruct-LUTS	hypertrophy (benign) of prostate with urinary obstruction and other lower urinary tract symptoms (LUTS)
6078	Other specified disorders of penis	Other specified disorders of penis
60784	impotence of organic origin	impotence of organic origin
61610		Vaginitis and vulvovaginitis unspecified

6259	unspecified symptom associated with female genital organs	unspecified symptom associated with female genital organs
6272		Symptomatic menopausal or female climacteric states
6929	unspecified inflamma of skin and subcutaneous	unspecified cause of inflammatory conditions of skin and subcutaneous tissue
6953	rosacea	rosacea
6961	other psoriasis	other psoriasis
7020	actinic keratosis	actinic keratosis
7021	Seborrheic keratosis	Seborrheic keratosis
70211	inflamed seborrheic keratosis	inflamed seborrheic keratosis
70219	other seborrheic keratosis	other seborrheic keratosis
7061	other acne	other acne
7062	sebaceous cyst	sebaceous cyst
7090	Dyschromia	Dyschromia
70909	other dyschromia	other dyschromia
7099	unspecified disorder of skin and subcutaneous tissue	unspecified disorder of skin and subcutaneous tissue
7140		Rheumatoid arthritis
7159	Osteoarthrosis: unspecified whether generalized or localized	Osteoarthrosis: unspecified whether generalized or localized
71590	osteoarthrosis: unspecified	osteoarthrosis: unspecified whether generalized or localized
71596	osteoarthrosis: lower leg	osteoarthrosis: lower leg
7169	unspecified arthropathy	unspecified arthropathy
71690	site unspecified arthropathy	site unspecified arthropathy
7194	Pain in joint	Pain in joint
71941	pain in joint in shoulder region	pain in joint in shoulder region
71946	pain in joint of lower leg	pain in joint of lower leg
7231	cervicalgia (pain in neck)	cervicalgia (pain in neck)
7234		Brachia neuritis or radiculitis NOS
7242	lumbago	lumbago (other and unspecified disorders of back)
7244	thoracic or lumbosacral neuritis or radiculitis: unspecified	thoracic or lumbosacral neuritis or radiculitis: unspecified
7245	unspecified backache	unspecified backache
7291	myalgia and myositis: unspecified	myalgia and myositis: unspecified

7295	pain in limb	pain in limb
73300		Osteoporosis unspecified
78039		Other convulsions
7804	dizziness and giddiness	dizziness and giddiness
7805	Sleep disturbances	Sleep disturbances
78052	unspecified insomnia	unspecified insomnia
78057		Unspecified sleep apnea
7807	Malaise and fatigue	Malaise and fatigue
78079	other malaise and fatigue	other malaise and fatigue
7810	abnormal involuntary movements	abnormal involuntary movements
7820	disturbance of skin sensation	disturbance of skin sensation
7821		Rash and other nonspecific skin eruption
7823	edema	edema
7840	headache	headache
7851	palpitations (awareness of heart beat)	palpitations (awareness of heart beat)
7860	Dyspnea and respiratory abnormalities	Dyspnea and respiratory abnormalities
78605	shortness of breath	shortness of breath
78609	other dyspnea and respiratory abnormalities	other dyspnea and respiratory abnormalities
7862	cough	cough
7865	Chest pain	Chest pain
78650	unspecified chest pain	unspecified chest pain
7882	Retention of urine	Retention of urine
78820	retention of urine: unspecified	retention of urine: unspecified
7883	Urinary incontinence	Urinary incontinence
78830	urinary incontinence: unspecified	urinary incontinence: unspecified
7884	Frequency of urination and polyuria	Frequency of urination and polyuria
78841	urinary frequency	urinary frequency
7890	Abdominal pain	Abdominal pain
78900	abdominal pain: unspecified site	abdominal pain: unspecified site
7909	Other nonspecific findings on examination of blood	Other nonspecific findings on examination of blood
79093	elevated PSA	elevated prostate specific antigen
7962		Elevated blood pressure reading without diagnosis of hypertension

7999		Other unknown and unspecified cause
8470		Sprains and strains of other and unspecified parts of back-Neck
V108	Personal history of malignant neoplasm of other sites	Personal history of malignant neoplasm of other sites
V1083	other malignant neoplasm of skin	other malignant neoplasm of skin
V1249		Other disorders of nervous system and sense organs
V202	routine infant or child health check	routine infant or child health check
V221	supervision of other normal pregnancy	supervision of other normal pregnancy
V238	Other high-risk pregnancy	Other high-risk pregnancy
V2389	other high-risk pregnancy	other high-risk pregnancy
V431	Lens influence health	persons with a condition influencing their health status (Lens)
V456	States following surgery of eye and adnexa	States following surgery of eye and adnexa
V4561	cataract extraction status	cataract extraction status
V458	Other postprocedural status	Other postprocedural status
V4589	other postprocedural status	other postprocedural status
V508	other elective surgery	other elective surgery for purposes other than remedying health states
V583		Attention to dressings and sutures
V586	Long-term (current) drug use	Long-term (current) drug use
V5869	long-term (current) use of other medications	long-term (current) use of other medications
V588	Other specified procedures and aftercare	Other specified procedures and aftercare
V5883		Encounter for therapeutic drug monitoring
V5889	other specified aftercare	other specified aftercare
V6549		Other specified counseling
V670	Following surgery	Following surgery
V6700	following surgery exam: unspecified	following surgery examination: unspecified
V6709	following up examinations: other surgery	following up examinations: other surgery
V675	Following other treatment	Following other treatment
V6759	other follow up examination	other follow up examination
V679	unspecified follow-up examination	unspecified follow-up examination
V700	routine medical exam	routine general medical examination at a health care facility
V7189		Other specified suspected conditions
V720		Examination of eyes and vision
V723	Gynecological examination	Gynecological examination

V7231	routine gynecological exam	routine gynecological examination
V7647		Special screening for malignant neoplasms-vagina
V99		
V990		
V997		
