# Supplementary material for: A Visual Analytic Tool (VIADS) to Assist the Hypothesis Generation Process in Clinical Research: Mixed Methods Usability Study
Source: JMIR Hum Factors. 2023 Apr 27;10:e44644. doi: 10.2196/44644 (PMC10176142; doi:10.2196/44644)
Supplement: Multimedia Appendix 4 [file humanfactors_v10i1e44644_app4.pdf]

VIADS

a **Visual Interactive Analysis** tool for filtering and summarizing large **Data Sets** coded with hierarchical terminologies

VIADS study session-training

2021-12-02 @Clemson

Background

VIADS

a **Visual Interactive Analysis** tool for filtering and summarizing large **Data Sets** coded with hierarchical terminologies

- Visualization
  - Versus excel files
  - Hierarchical relationships
- Data sets coded by hierarchical ***terminologies*** in health care
  - ICD9 (monohierarchic)
  - ICD10 (monohierarchic)
  - MeSH (polyhierarchic)
- However

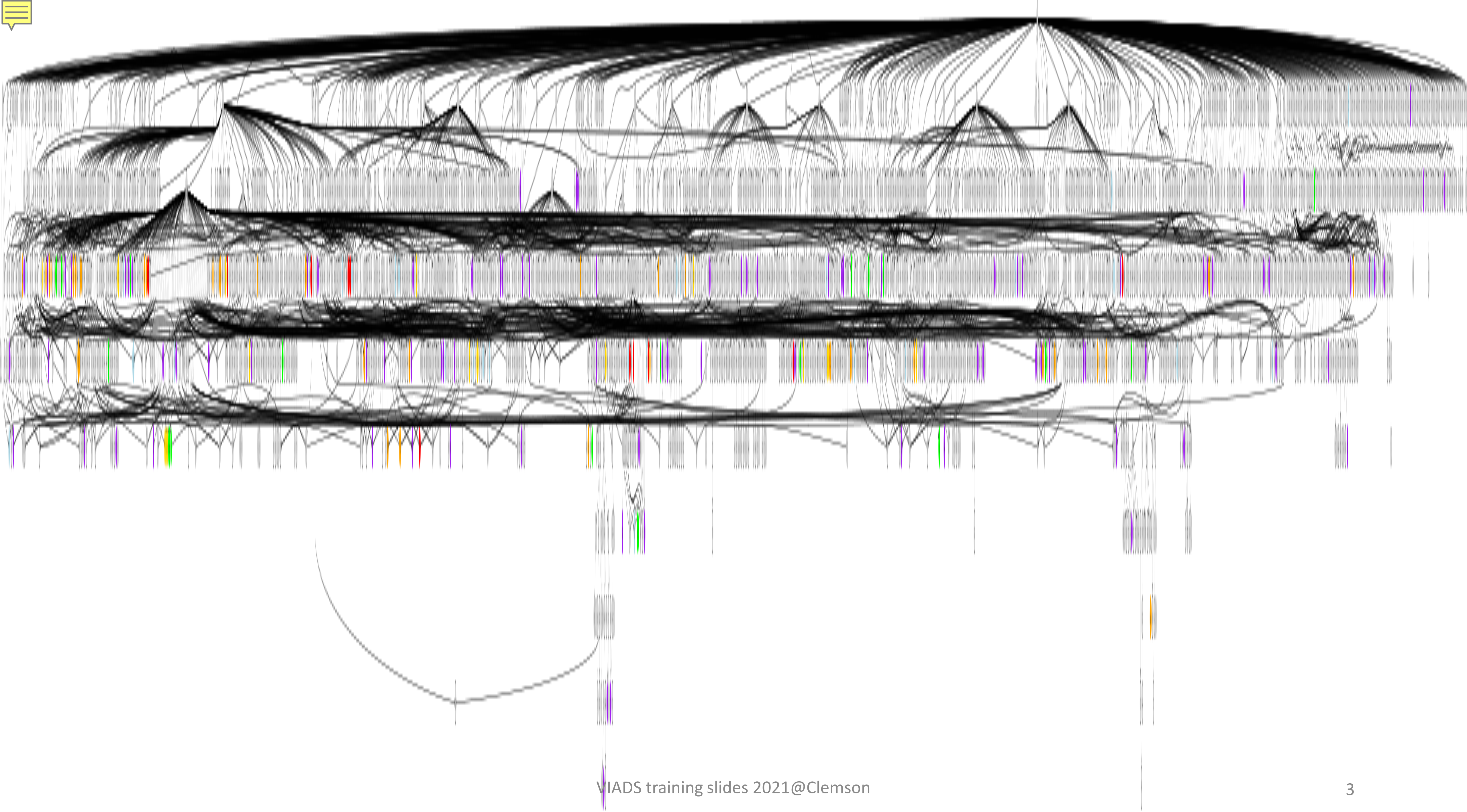

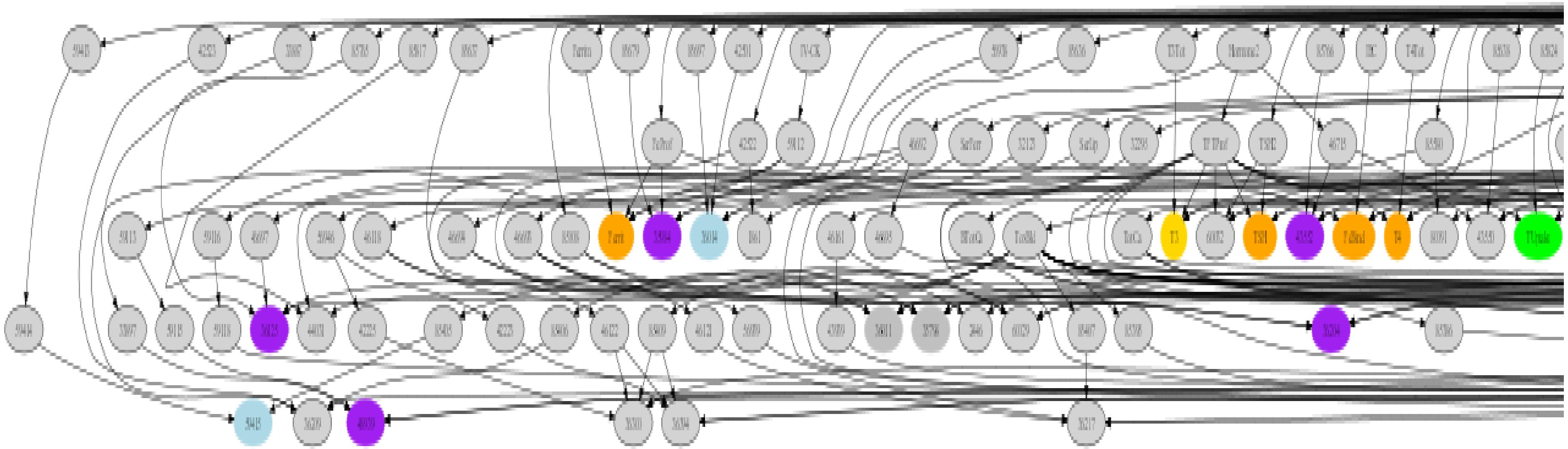

Background (Cont.)

VIADS

a **Visual Interactive Analysis** tool for filtering and summarizing large **Data Sets** coded with hierarchical terminologies

- Algorithms development
  - **Reduce** size
  - **Simplify** complexity
  - **Analyze** large data set
    - Summarize
    - Compare & highlight

  - By using hierarchical **terminologies** & **frequencies**

Filter --- **node counts (NC)**

If NC >= T_{nc}, then display node n.

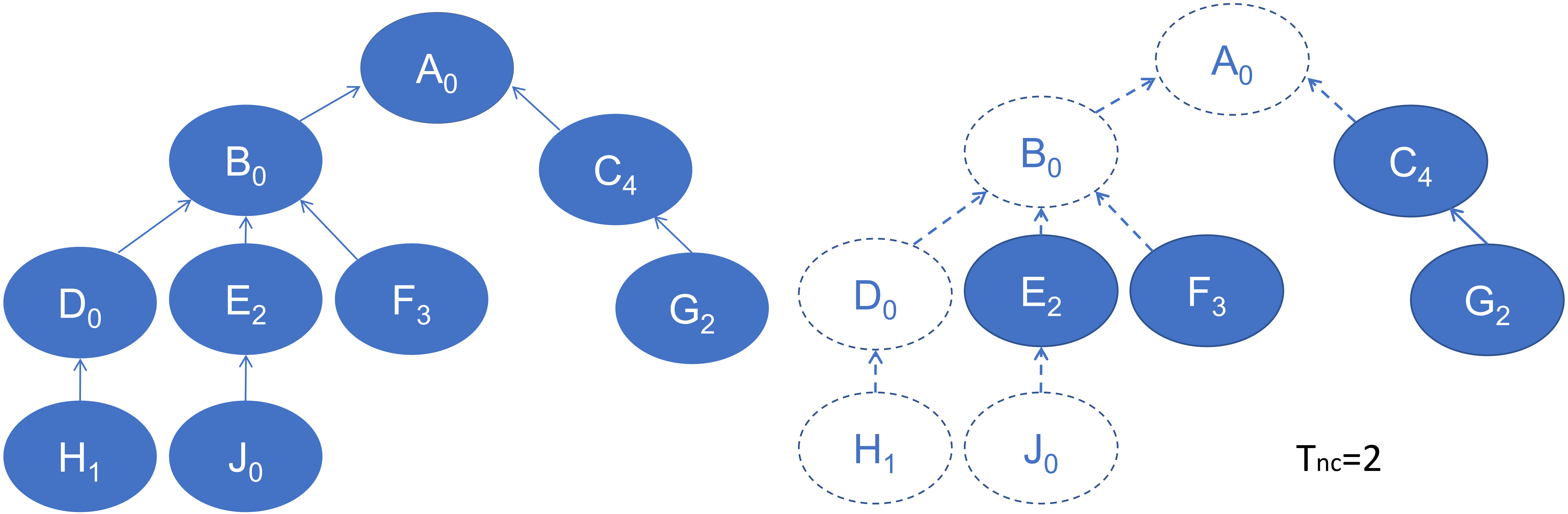

Filter --- class counts (CC)

- $CC_n = NC_{dn1} + NC_{dn2} + NC_{dn3} \ldots NC_{dnk}$

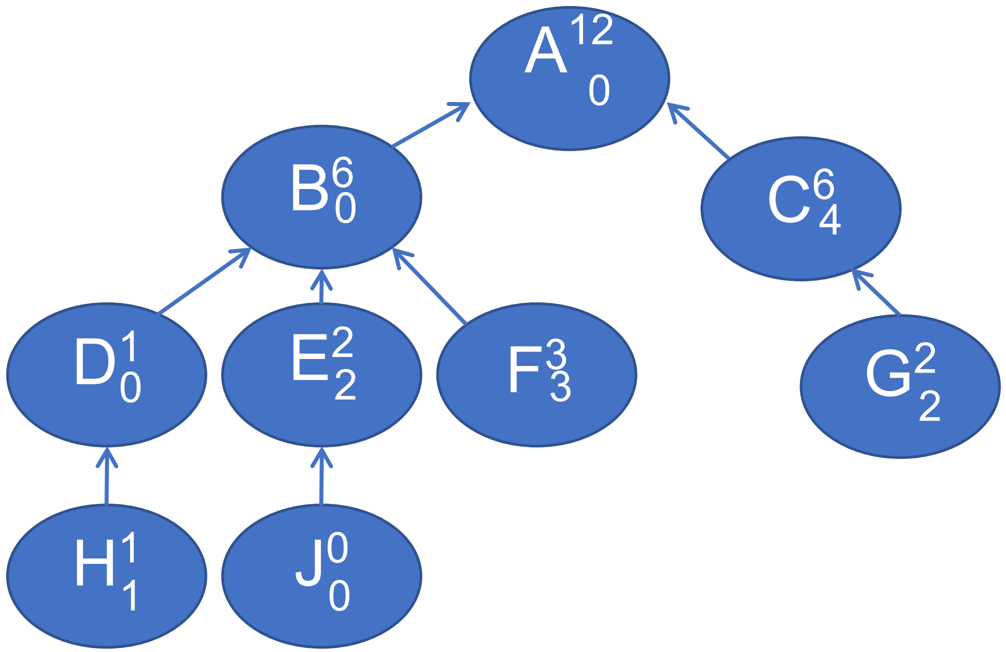

Table 1 Data table

Node	Node count	Class count
A	0	12
B	0	6
C	4	6
D	0	1
E	2	2
F	3	3
G	2	2
H	1	1
J	0	0

Filtering --- class counts

If CC_n >=T_{cc}, then display n and *all the ancestors* of n in the terminological structure.

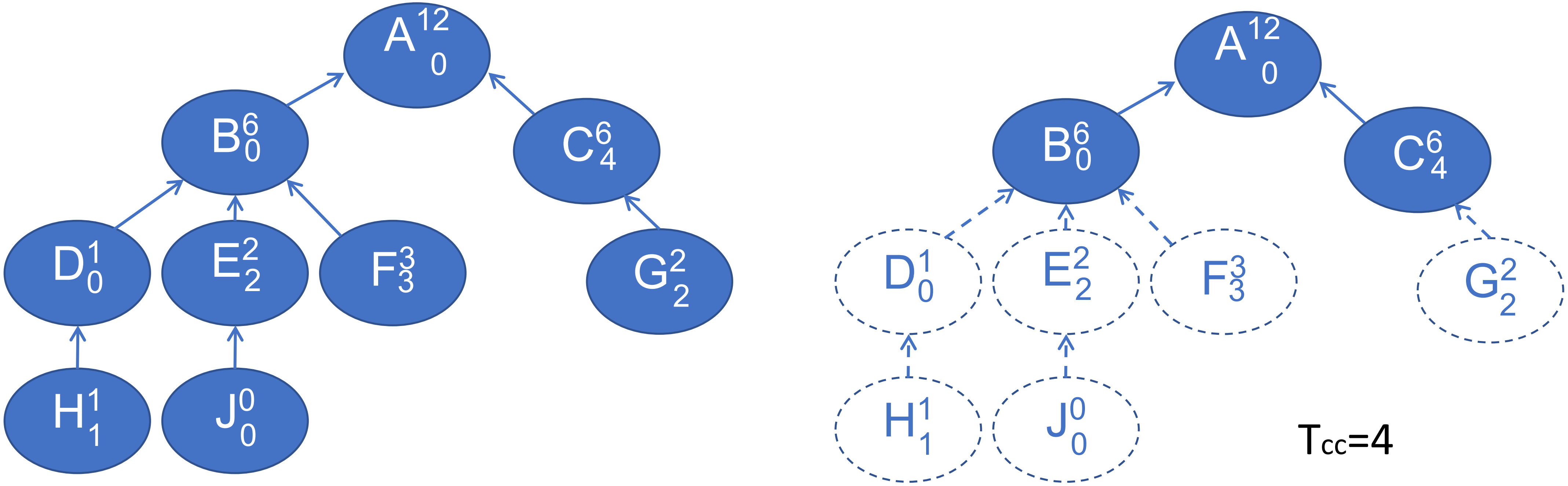

Filter --- **ratio values (RV)**

- $RV_n = CC_n / CC_p$

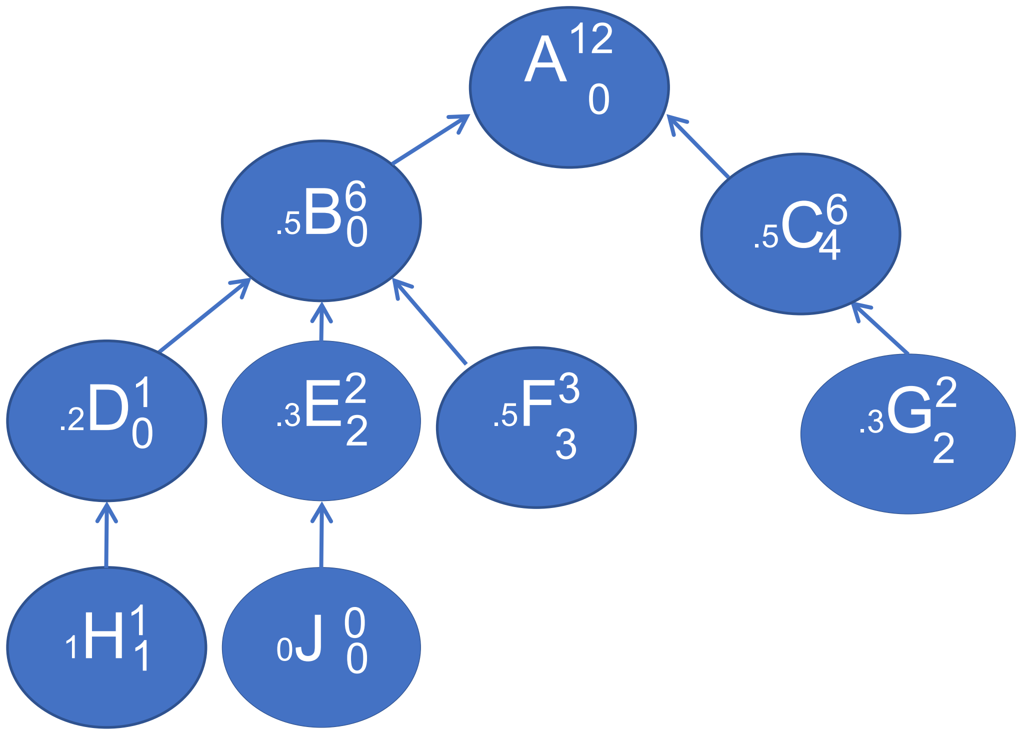

Table 1 Calculated ratio table

Node	CC	ratio
B	6	0.5
C	6	0.5
D	6	0.2
E	2	0.3
F	3	0.5
G	2	0.3
H	1	1
J	0	0

Filter --- **ratio values**

If $RV_n >= T_r$, then display n and all the ancestors of n in the terminological structure.

$RV_n = CC_n / CC_p$

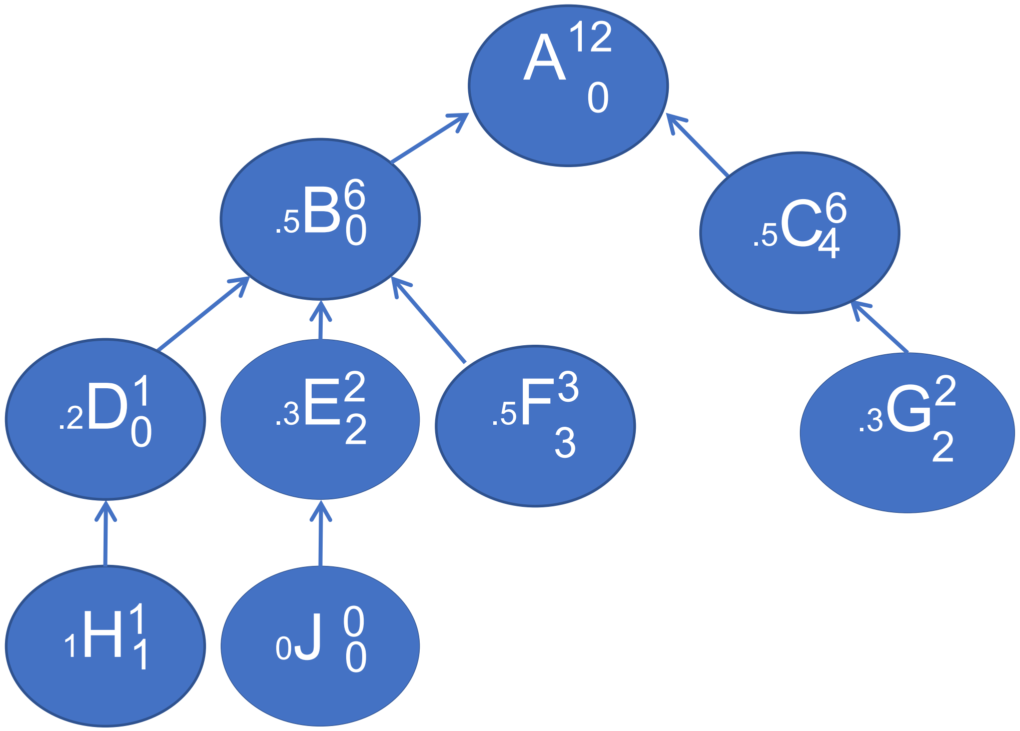

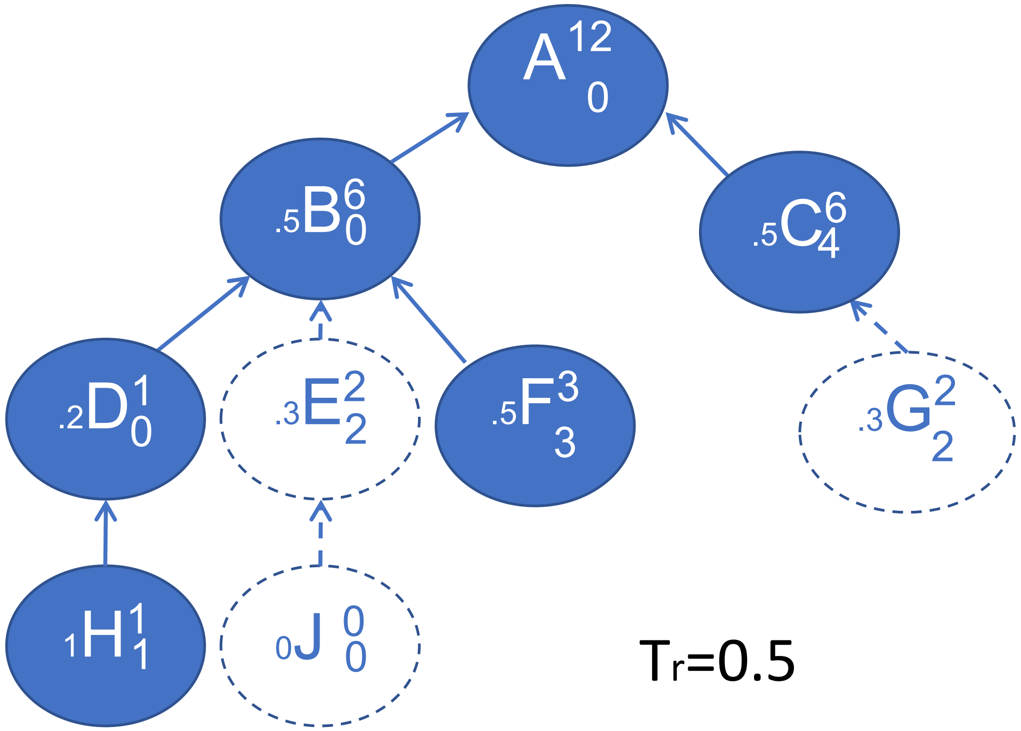

$T_r = 0.5$

Comparison of similar data sets(diff)

- Comparison of two similar data sets
  - Two decades, two similar medications (Actos, Avandia), before and after a procedure …

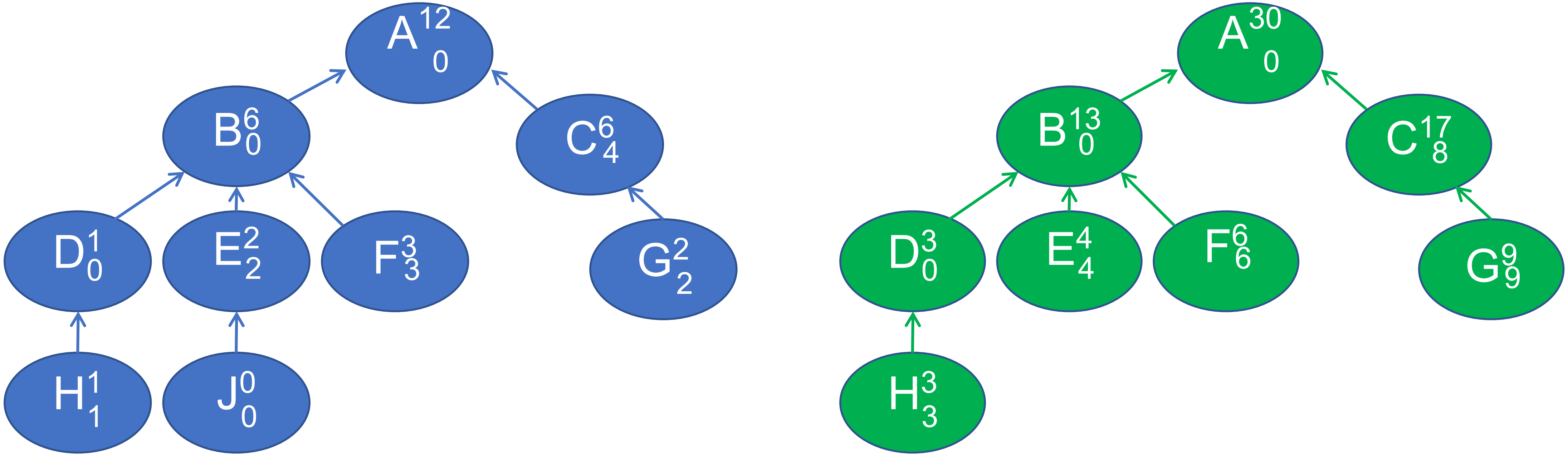

Filter --- p values for diff data sets (prop.test)

If $P_n >= T_p$, then display n and all the ancestors of n in the terminological structure.

$$CCP(term) = CC(term) / \sum_{k=1}^{n} CC(term)\ k$$

Node	CC in data set 1	CC in data set 2	P value
A	12	30	1
B	6 (6/12)	13 (13/30)	0.0065
C	6 (6/12)	17 (17/30)	0.0219
D	1 (1/12)	3 (3/30)	1
E	2 (2/12)	4 (4/30)	0.8521
F	3 (3/12)	6 (6/30)	0.6062
G	2 (2/12)	9 (9/30)	1
H	1 (2/12)	3 (3/30)	1

CC+RV --- thresholds setting

- 3D plot (scatter3dplot, R)- highlight points
  - CC, RV, the number of nodes in filtered graphs
- Regression plane
  - Minimizes the total of the squared distances: the observed values → the closest point on the regression plane
  - The least squared distance (residual, R)
- Select data points
  - **90-120** + **closest to the plane**

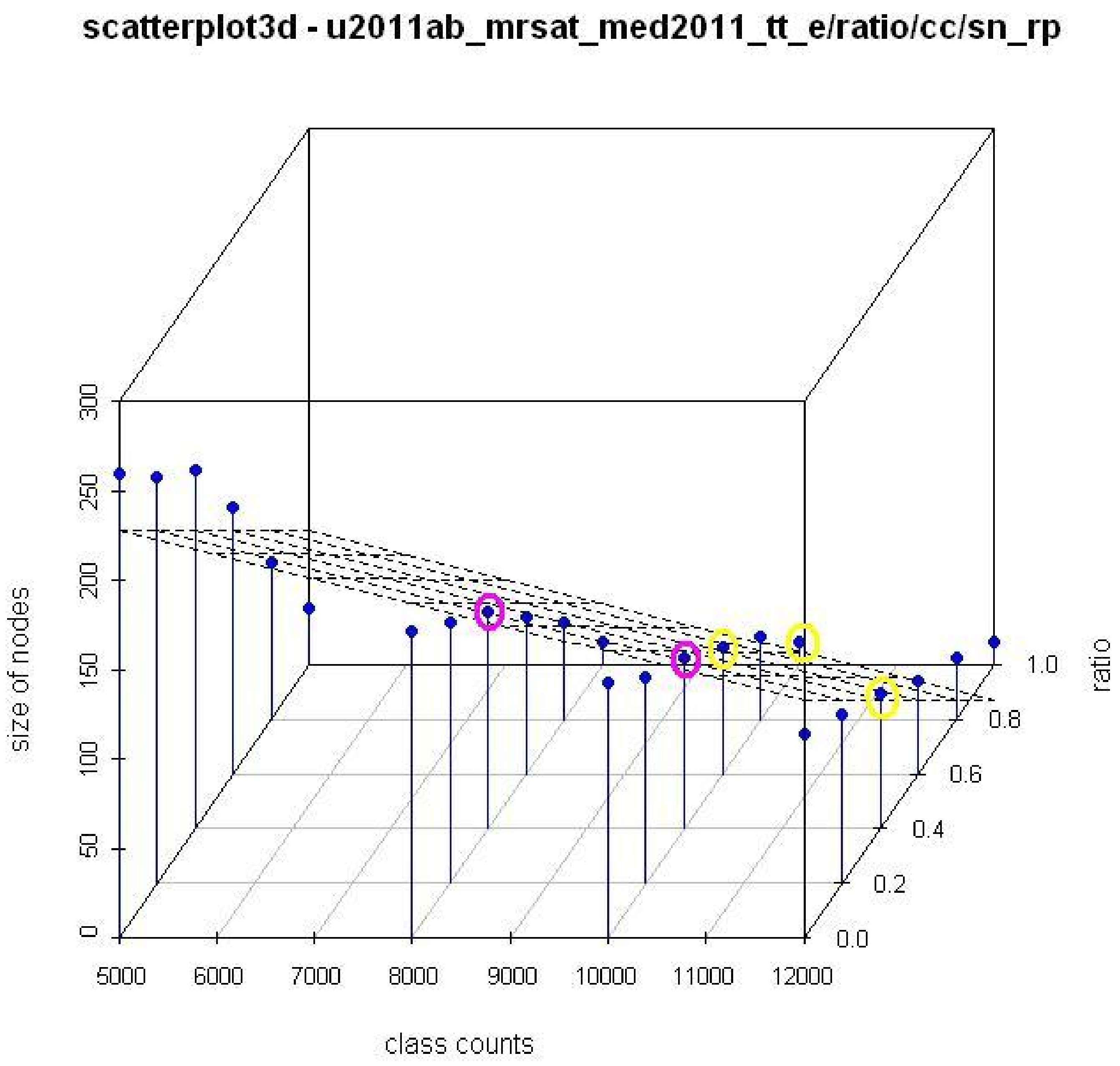

3D plot of class count thresholds, ratio value thresholds and the number of nodes in filtered graphs for MeSH terms in 2011 with the regression plane.

Purposes of using VIADS

- Facilitate to understand the data sets
- See the bigger picture, aggregated effects
- Highlight the differences
- By trimming down the relatively unimportant parts

- To generate *new research ideas*

Diseases and Injuries Tabular Index

1. INFECTIOUS AND PARASITIC DISEASES (001-139)
2. NEOPLASMS (140-239)
3. ENDOCRINE, NUTRITIONAL AND METABOLIC DISEASES, AND IMMUNITY DISORDERS (240-279)
4. DISEASES OF THE BLOOD AND BLOOD-FORMING ORGANS (280-289)
5. MENTAL DISORDERS (290-319)
6. DISEASES OF THE NERVOUS SYSTEM AND SENSE ORGANS (320-389)
7. DISEASES OF THE CIRCULATORY SYSTEM (390-459)
8. DISEASES OF THE RESPIRATORY SYSTEM (460-519)
9. DISEASES OF THE DIGESTIVE SYSTEM (520-579)
10. DISEASES OF THE GENITOURINARY SYSTEM (580-629)
11. COMPLICATIONS OF PREGNANCY, CHILDBIRTH, AND THE PUERPERIUM (630-679)
12. DISEASES OF THE SKIN AND SUBCUTANEOUS TISSUE (680-709)
13. DISEASES OF THE MUSCULOSKELETAL SYSTEM AND CONNECTIVE TISSUE (710-739)
14. CONGENITAL ANOMALIES (740-759)
15. CERTAIN CONDITIONS ORIGINATING IN THE PERINATAL PERIOD (760-779)
16. SYMPTOMS, SIGNS, AND ILL-DEFINED CONDITIONS (780-799)
17. INJURY AND POISONING (800-999)

SUPPLEMENTARY CLASSIFICATION OF FACTORS INFLUENCING HEALTH STATUS AND CONTACT WITH HEALTH SERVICES (V01-V89)

SUPPLEMENTARY CLASSIFICATION OF EXTERNAL CAUSES OF INJURY AND POISONING (E800-E999)

1. INFECTIOUS AND PARASITIC DISEASES (001-139)

TUBERCULOSIS (010-018)

Includes:

infection by Mycobacterium tuberculosis (human) (bovine)

Excludes:

congenital tuberculosis (771.2)

late effects of tuberculosis (137.0-137.4)

The following fifth-digit subclassification is for use with categories 010-018:

0 unspecified

1 bacteriological or histological examination not done

2 bacteriological or histological examination unknown (at present)

3 tubercle bacilli found (in sputum) by microscopy

4 tubercle bacilli not found (in sputum) by microscopy, but found by bacterial culture

5 tubercle bacilli not found by bacteriological examination, but tuberculosis confirmed histologically

6 tubercle bacilli not found by bacteriological or histological examination, but tuberculosis confirmed by other methods [inoculation of animals]

- **010 Primary tuberculous infection**

  Requires fifth digit. See beginning of section 010-018 for codes and definitions.

- **011 Pulmonary tuberculosis**

  Requires fifth digit. See beginning of section 010-018 or codes and definitions.

  Use additional code to identify any associated silicosis (502)

- **012 Other respiratory tuberculosis**

  Requires fifth digit. See beginning of section 010-018 for codes and defini

  Excludes:

  respiratory tuberculosis, unspecified (011.9)

- **013 Tuberculosis of meninges and central nervous system**

  Requires fifth digit. See beginning of section 010-018 for codes and defini

TUBERCULOSIS (010-018)

010 Primary tuberculous infection

Requires fifth digit. See beginning of section 010-018 for codes and definitions.

010.0 Primary tuberculous infection

[0-6]

Excludes:

nonspecific reaction to tuberculin skin test without active tuberculosis (795.5)

positive PPD (795.5)

positive tuberculin skin test without active tuberculosis (795.5)

010.1 Tuberculous pleurisy in primary progressive tuberculosis

[0-6]

010.8 Other primary progressive tuberculosis

[0-6]

Excludes:

tuberculous erythema nodosum (017.1)

010.9 Primary tuberculous infection, unspecified

[0-6]
