# Supplementary material for: A Visual Analytic Tool (VIADS) to Assist the Hypothesis Generation Process in Clinical Research: Mixed Methods Usability Study
Source: JMIR Hum Factors. 2023 Apr 27;10:e44644. doi: 10.2196/44644 (PMC10176142; doi:10.2196/44644)
Supplement: Multimedia Appendix 5 [file humanfactors_v10i1e44644_app5.pdf]

VIADS USER MANUAL

Summary: VIADS (visual interactive analysis tool for filtering and summarizing large data sets coded with hierarchical terminologies, https://www.viads.info) is a tool that summarizes, analyzes, filters, and visualizes large data sets that coded with hierarchical terminologies, such as ICD-10 or MeSH. It is an online publicly accessible data analytic tool, which is free for educational and research purposes. VIADS accommodates guest users and permanent registered users.

Login System

Account Types

There are two account types, **registered accounts** and **guest accounts**. Registered accounts allow the user to return to the website and use the files they've previously created and analyzed. Guest accounts allow the user to use the website, but the results cannot be retrieved after the user has closed the browser or been on the site for over 24 hours.

Registered Accounts

To create a registered account, click the Create Account link on the homepage. Of the fields listed, only ***username***, ***email***, ***password***, and ***password confirmation*** are required. Passwords must be at least eight characters, must not be entirely numeric, and cannot be a common password(e.g., password). Once a user has entered in all the required information, click the "I'm not a robot" box, press submit, and an email will be sent to the user's email address with an activation link. The account cannot be used until the account is activated. Once a registered user logs in, the user will be able to use any files they have previously uploaded/saved.

Guest Accounts

To create a guest account, click the Guest Account link on the homepage. Click the "I'm not a robot box" and submit. Guest accounts are only active for a maximum of 24 hours, and cannot be returned to once you have exited your browser. Guest accounts still allow users to fully utilize other functionalities within the website.

Dashboard

The dashboard is only functional if the user has **logged in**, or is using a **guest account**.

The Tables

There are three tables which are presented to the user. Only one table is shown at a time, you must click on the boxes on the left to cycle between which one is presented to you.

Clicking on a **column header** in one of the tables sorts the files in the table by that parameter. Currently, the most recently sorted parameter has its column header colored in VIADS blue. It will soon be changed to use **arrows** to indicate sorting.

Right Buttons

On the right is various links to other features of the VIADS website, for example, Upload File, Analyze, Download. It should be noted that some of the options are displayed and hidden based on which **table** is currently being viewed. For instance, the user can not select the option to analyze an output file - only input files can be analyzed.

The **Download**, **Log Out** and **My Account** links can be found on the right regardless of which table is being viewed.

Validation

The validation module begins when you click “Upload File” in the dashboard

File Select

Upon selecting “Upload File” you will be sent to the file select page. There are a few options here. You need to select the ***terminology*** that coded your dataset: ICD9-CM, ICD10-CM, or MeSH. Then you may choose whether your data uses ***subject counts or event counts***. Also, you need to choose the ***cell separator*** in your CSV file. Most CSVs use commas as their cell separator, so use this if you are not sure. Some CSVs use pipes, and if you have another specific separator you can select custom and enter it.. These details should be provided to you from where you received your dataset.

Click “Choose file” to navigate to where your file is locally on your computer. In the text area you may choose a name for the file to be saved as, otherwise a default file name will be assigned to the file you uploaded.

When you are ready click “Upload” to continue.

Validation

If there are errors in the uploaded data set, you will be prompted to either click “continue” to try to resolve these errors or click “abort” to return to the dashboard. If there were no errors continue to the next section.

If you click “continue”, the validation module will guide you through fixing errors in the uploaded dataset. You will be prompted with a select box that lets you choose the type of fix, and sometimes a text box to enter needed information. There will sometimes be a checkbox labeled “Do for all errors of this type” which will attempt to apply the select fix for all errors of the same time in this dataset. Possible errors include:

- Invalid code - This shows that the code you supplied was invalid. You may either remove the entry for this code or rename the code. Please look up the source and terminology for your dataset for details
- Duplicate code - The same code appears multiple times in your dataset. This error does not apply to MeSH codes. You may:
    - Choose to set the code manually, and all instances of this code in your dataset will be deleted and a new entry will be added for it with the specified frequency

- Remove all instances of this code in your dataset
    - Average the frequencies for this code, and set the average as the frequency for the specified code
    - Sum the frequencies for this code, and set the sum as the frequency for the specified code
    - Choose one record (instance) to keep, and all other instances of this code will be removed from your dataset
- Line is too long - A line from the dataset has more than two columns. You may:
    - Remove this record (instance)
    - Manually change this record to a specified code and frequency
    - Trim off the extra cells after the first two columns
- Code is not in parent child table
    - If the code's parent is in the default parent child table, or its grandparent is, you will be given the option to to add an entry to the parent child code for this code, otherwise you only may delete that code from the dataset
- Code has an invalid frequency - The frequency of a code must be a positive integer. Fix options include:
    - Removing the entry/record
    - Manually setting the frequency
    - If frequency is a positive number but not an integer, you have the option to round it to the nearest integer
- Frequency low - For this error, the sum of all of the frequencies is too low to be analyzed. For example: the minimal acceptable aggregated frequency is 100 for subject counts and 1000 for event counts. Please upload a larger dataset

Once all errors are fixed, you will be shown a dialog saying "No errors were found in the uploaded dataset." From here you can either abort back to the dashboard or you can continue to the file summary page. If you click continue your file will be saved and you will be presented with information about the validation summary of your file and then the option to return to the dashboard

Visualization

Single Graph Analysis

After selecting your validated dataset from the dashboard and selecting **Analyze**, you will be brought to a new page where you will be presented with a control panel. You may return to the dashboard at any time by clicking the **Return To Dashboard** button in the top left of the control panel.

The following section will describe each of the options presented:

Algorithm
These radio buttons will allow you to select the algorithm you wish to use.

NC (node count)

This algorithm displays all nodes with frequencies greater than or equal to the given threshold.

CC (class count)
This algorithm displays all nodes with aggregated frequencies greater than or equal to the given threshold

NC+RATIO
This algorithm will display all nodes that have frequencies greater than or equal to the given threshold and whose aggregated frequency contributes a ratio of the parent node's aggregated frequency that is greater than or equal to the given ratio.

CC+RATIO
This algorithm will display all nodes that have aggregated frequencies greater than or equal to the given threshold and whose aggregated frequency contributes a ratio of the parent node's aggregated frequency that is greater than or equal to the given ratio.

TOP NC
This algorithm will display the top N nodes sorted by frequency where N is the number given by the user.

TOP CC
This algorithm will display the top N nodes sorted by aggregated frequency where N is the number given by the user.

NC %
This algorithm will display the top N percent of nodes sorted by frequency where N is the number given by the user.

CC%
This algorithm will display the top N percent of nodes sorted by frequency where N is the number given by the user.

Tune threshold
This is where the user will enter thresholds to test against. The title of the input boxes may change when selecting new algorithms. For algorithms that use ratios, the user will need to define both a frequency and a ratio threshold.

Calculate Number Of Nodes
This button will calculate how many nodes will appear when a graph is generated with the given threshold. This button is useful ensuring that the user does not accidentally try to render a graph with too many nodes to process.

Generate From Data

This button will generate a graph in the area below the control panel.

Results Preview
Depending on the algorithm, this button will generate either a 2D bar graph or a 3D scatter plot. The data points of the generated graphs show the user how many nodes they can expect to be returned in a generated graph when using different values.

Many of the previews select thresholds that will ensure a reasonable amount of nodes to be returned.

Save
After you have input a name for the file, this button will allow the user to save the output of a test he/she has ran for later viewing. Saved graphs can be viewed in the dashboard.

Download Graph File
This link will download a .csv file containing all of the important information of each node in a generated graph.

Download SVG
This link will download a .svg image of the generated graph.

Download PNG
This link will download a .png image of the generate graph.

Graph Comparison Analysis

After selecting two validated datasets from the dashboard and selecting **Compare**, you will be brought to a new page where you will be presented with a control panel. Note that the selected data sets must have a matching code structure (ICD9 & ICD9, ICD10 & ICD10, MeSH & MeSH).You may return to the dashboard at any time by clicking the **Return To Dashboard** button in the top left of the control panel

The functionality of this control panel is similar to the control panel for analyzing a single graph. Please refer to the instructions for single graph analysis for information on how the control panel works. The algorithm presented, differs from the algorithm used for single dataset analysis

CC comparison
This algorithm runs a proportion test on the nodes from both data sets using the node aggregated frequencies as data points. The nodes returned will be those which had P-values under the given threshold.

Manipulating the Graph

The generated graph can be interacted with in a few ways:

Horizontal Distance
These radio buttons will allow the user to change the distance between the nodes in the graph. Useful if the graph has many nodes and the connecting edges are cluttered.

Arrow
The arrow at the bottom of the control panel will allow the user to collapse the control panel to make more room for viewing the generated graph. To get the control panel back, simply click the arrow again.

Zoom In/Out
These buttons will zoom in or out of the generated graph. These buttons are primarily for users without a mouse wheel. The graph can also be zoomed by scrolling with the mouse.

Key
Hovering over the **Key** in the bottom right corner will display a graphical key explaining the algorithms and the meaning of the colors in the generated graph. To hide the **Key** again, simply move the mouse off the key.

Panning
To pan the view, click and drag the background of the graph.

Zooming
To zoom in or out, scroll with the mouse wheel or use the zoom buttons in the lower left corner.

Selecting Nodes
Clicking on a node will highlight that node and also all of the connected edges.

Rearranging Nodes
The user can move nodes around by clicking and dragging them. The nodes use a physics simulation to compact themselves into a small area which may be slow on older computers or when generating a graph with a large number of nodes. By default, the program attempts to place the nodes in an order that keeps things as free as possible of clutter.
